# Inhibition of Respiration of Candida albicans by Small Molecules Increases Phagocytosis Efficacy by Macrophages

**DOI:** 10.1128/mSphere.00016-20

**Published:** 2020-04-15

**Authors:** Shuna Cui, Minghui Li, Rabeay Y. A. Hassan, Anna Heintz-Buschart, Junsong Wang, Ursula Bilitewski

**Affiliations:** a Jiangsu Key Laboratory of Integrated Traditional Chinese and Western Medicine for Prevention and Treatment of Senile Diseases, Medical College of Yangzhou University, Yangzhou, China; b Helmholtz Centre for Infection Research (HZI), Braunschweig, Germany; c Department of Obstetrics and Gynecology, Affiliated Hospital of Yangzhou University, Yangzhou, China; d Jiangsu Co-Innovation Center for Prevention and Control of Important Animal Infectious Diseases and Zoonoses, College of Veterinary Medicine, Yangzhou, China; e School of Environmental and Biological Engineering, Nanjing University of Science and Technology, Nanjing, China; f Center for Materials Sciences, Zewail City of Science and Technology, Giza, Egypt; g Applied Organic Chemistry Department, National Research Centre (NRC), Giza, Egypt; h Department of Soil Ecology, Helmholtz-Centre for Environmental Research GmbH—UFZ, Halle/Saale, Germany; i German Centre for Integrative Biodiversity Research (iDiv) Halle-Jena-Leipzig, Leipzig, Germany; University of Georgia

**Keywords:** complex III, respiratory chain, antimycin A, phagocytosis, metabolism

## Abstract

Candida albicans adapts to various conditions in different body niches by regulating gene expression, protein synthesis, and metabolic pathways. These adaptive reactions not only allow survival but also influence the interaction with host cells, which is governed by the composition and structure of the fungal cell wall. Numerous studies had shown linkages between mitochondrial functionality, cell wall integrity and structure, and pathogenicity. Thus, we decided to inhibit single complexes of the respiratory chain of C. albicans and to analyze the resultant interaction with macrophages via their phagocytic activity. Remarkably, inhibition of the fungal bc_1_ complex by antimycin A increased phagocytosis, which correlated with an increased accessibility of β-glucans. To contribute to mechanistic insights, we performed metabolic studies, which highlighted significant changes in the abundance of constituents of the plasma membrane. Collectively, our results reinforce the strong linkage between fungal energy metabolism and other components of fungal physiology, which also determine the vulnerability to immune defense reactions.

**IMPORTANCE** The yeast Candida albicans is one of the major fungal human pathogens, for which new therapeutic approaches are required. We aimed at enhancements of the phagocytosis efficacy of macrophages by targeting the cell wall structure of C. albicans, as the coverage of the β-glucan layer by mannans is one of the immune escape mechanisms of the fungus. We unambiguously show that inhibition of the fungal bc_1_ complex correlates with increased accessibilities of β-glucans and improved phagocytosis efficiency. Metabolic studies proved not only the known direct effects on reactive oxygen species (ROS) production and fermentative pathways but also the clear downregulation of the ergosterol pathway and upregulation of unsaturated fatty acids. The changed composition of the plasma membrane could also influence the interaction with the overlying cell wall. Thus, our work highlights the far-reaching relevance of energy metabolism, indirectly also for host-pathogen interactions, without affecting viability.

## Abstract (precis)

The yeast Candida albicans is one of the major fungal human pathogens, for which new therapeutic approaches are required. We aimed at enhancements of the phagocytosis efficacy of macrophages by targeting the cell wall structure of C. albicans, as the coverage of the β-glucan layer by mannans is one of the immune escape mechanisms of the fungus. We unambiguously show that inhibition of the fungal bc_1_ complex correlates with increased accessibilities of β-glucans and improved phagocytosis efficiency. Metabolic studies proved not only the known direct effects on reactive oxygen species (ROS) production and fermentative pathways but also the clear downregulation of the ergosterol pathway and upregulation of unsaturated fatty acids. The changed composition of the plasma membrane could also influence the interaction with the overlying cell wall. Thus, our work highlights the far-reaching relevance of energy metabolism, indirectly also for host-pathogen interactions, without affecting viability.

## INTRODUCTION

Candida albicans asymptomatically colonizes mucosal surfaces of most healthy individuals. It turns into a pathogen when it can penetrate the physical barriers of the skin and mucosa, evades the attack from the immune system, and multiplies within the affected body niche. Thus, interactions between the fungus and host cells are decisive for the switch from a commensal organism to a pathogen. They are governed by pathogen-associated molecular pattern (PAMPs) of the fungal cell wall and the corresponding pattern recognition receptors (PRRs) of the mammalian cell membrane. The fungal cell wall can schematically be described as a layered structure of polysaccharides with bound proteins. Closest to the plasma membrane is chitin, a polymer of *N*-acetylglucosamine, followed by a network of β-1,3- and β-1,6-glucans and finally mannoproteins (1). The major immunostimulatory PAMPs of C. albicans are the β-glucans, particularly β-(1,3)-glucans. They are recognized by the C-type lectin-like receptor dectin-1, which induces phagocytosis of fungal pathogens and promotes the production of proinflammatory cytokines (2). In living C. albicans cells, the β-glucans are hidden under a mannoprotein coat, presumably as an immune escape mechanism. However, the cell wall structure is dynamic and responds to environmental conditions. Growth of C. albicans at acidic pH (3) or inactivation of the fungus by heat (4) leads to an increased exposure of the β-glucans, which is usually accompanied by increased phagocytic activity of macrophages via increased binding of the PRR dectin-1. Important signal transduction elements for adaptation to changing environments are the mitogen-activated protein kinases (MAPKs), among which the Cek1 MAPK mediates cell wall biogenesis. It was shown that disruption of the Cek1-mediated pathway (5), but also constitutive activation of Cek1 (6), promotes the exposure of β-1,3-glucan and, again, the binding of dectin-1 and phagocytosis by macrophages and dendritic cells. Similar effects were observed when genes for the histidine kinase CHK1 (4, 7) or for proteins required for mannosylation of cell wall proteins (8) were deleted. As the cell wall is connected to the plasma membrane via glycosylphosphatidylinositol (GPI)-anchored proteins, the plasma membrane composition influences the cell wall structure, and the reduced production of the phospholipid phosphatidylserine reduced the masking of β-glucans and increased binding of dectin-1 (9). However, also the application of sublethal concentrations of an inhibitor of β-(1,3)-d-glucan synthase, caspofungin (8), and of respiratory chain inhibitors (10) led to rearrangements of the cell wall, which increased the uptake by phagocytes. On the other hand, growth under hypoxic conditions triggered β-glucan masking and concomitantly reduced phagocytosis (11). Thus, there is also a link between the oxidative metabolism of the fungus, which occurs mainly in mitochondria, and the cell wall structure. This is in line with the observation that the deletion of genes affecting the activity of the mitochondrial complex I influenced the cell wall structure. Deletion of the regulator of complex I, *GOA1* (12), but also of subunit proteins, such as Nuo1 and Nuo2 (13), led to reduced recognition by immune cells, probably via the decreased expression of genes required for biosynthesis of cell wall mannans, but also of membrane constituents, such as phospholipids and ergosterol.

However, the existing studies are focused on fungus-specific complexes of the respiratory chain, such as the alternative oxidases (10, 14), or on the link to morphology (15), the major virulence factor of C. albicans. The role of components of the classical respiratory chain in the cell wall structure and, thus, in the interaction with host cells, is yet largely underexplored.

Therefore, we decided to treat C. albicans with specific inhibitors of the major complexes I to IV of the electron transport chain and to determine the phagocytic efficiency of macrophages for these pretreated fungal cells. We chose rotenone, thenoyltrifluoroacetone (TTFA), antimycin A (AA), and potassium cyanide (KCN) as specific inhibitors of complexes I to IV, and salicylhydroxamic acid (SHA) as an inhibitor of alternative oxidases. We observed an enhancement of phagocytosis, in particular after C. albicans was treated with AA, which correlated with an increased exposure of β-(1,3)-glucans and a redirection of metabolic pathways. Thus, our results highlight the specific role of complex III of the classical mitochondrial electron transport chain for the adaptation of cell wall structure and cell membrane composition.

## RESULTS

### Treatment of C. albicans with AA increased phagocytic efficiency of the macrophage cell line RAW 264.7.

**FIG 1:**
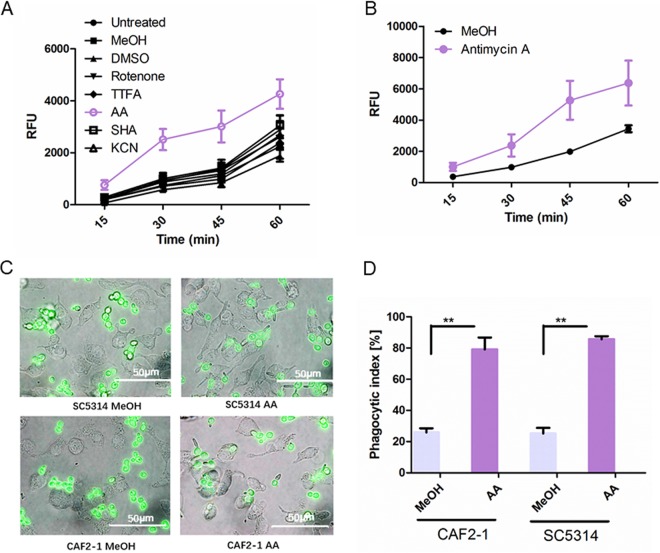
AA treatment strongly enhances phagocytosis of C. albicans. (A) Phagocytic activity of macrophages (RAW 264.7) against C. albicans SC5314 after overnight treatment with respiratory chain inhibitors by a fluorimetric assay. RFU, relative fluorescence units. Concentrations of respiratory chain inhibitors in these experiments: rotenone, 40 mg/liter dissolved in DMSO; TTFA, 5 mg/liter; SHA, 50 mg/liter; antimycin A, 0.5 mg/liter (all dissolved in MeOH); KCN, 5 mg/liter dissolved in water. (B) Phagocytic activity of RAW 264.7 macrophage cell line against C. albicans CAF2-1 after overnight treatment with AA. Data shown are representative of at least three independent experiments. Mean values and standard deviations were calculated from at least six biological replicates in each experiment. (C) Representative microscopic pictures of C. albicans SC5314 and CAF2-1 ingested by the murine macrophage cell line RAW 264.7. C. albicans was labeled with FITC, which is shown in the picture in green fluorescence (60×). The picture was taken after 1 h of infection. (D) Phagocytic indices of AA-treated C. albicans strains SC5314 and CAF2-1. The histogram quantitatively compares the percentage of the number of macrophages with intracellular yeasts to the total number of macrophages per picture. Data are based on the means for at least three independent experiments. **, *P* < 0.01.

C. albicans strain SC5314 was grown in the presence of the respiratory chain inhibitors rotenone (40 mg/liter) (complex I), thenoyltrifluoroacetone (5 mg/liter) (TTFA, complex II), antimycin (0.5 mg/liter) (AA, bc_1_ complex), salicylhydroxamic acid (50 mg/liter) (SHA, alternative oxidase [AOX]), and potassium cyanide (5 mg/liter) (KCN, complex IV). The concentrations were chosen according to previous studies (15) and mainly based on inhibitory effects on oxygen consumption and reactive oxygen species (ROS) induction. The murine macrophage cell line RAW 264.7 was infected with the treated fungal cells and showed significantly enhanced phagocytosis when C. albicans SC5314 was treated with AA. Inhibitors of the other complexes of the respiratory chain had almost no effect on the phagocytic activity (Fig. 1A). Thus, the following investigations were focused on the effects resulting from the inhibition of complex III.

As a number of C. albicans single gene deletion mutants, which are used in other studies, were derived from C. albicans CAF2-1, we also used this strain in our experiments (Fig. 1B). It showed the same response as the wild-type strain SC5314. All following experiments were done with CAF2-1.

To validate the results from the fluorimetric microplate phagocytosis assay, we evaluated the ingestion of C. albicans treated with AA by fluorescence microscopy (Fig. 1C). When C. albicans was treated with the solvent methanol (MeOH), a rather high number of macrophages were found without ingested yeast cells; when the fungus was treated with AA, the ratio of ingested yeast cells increased. This visual impression was confirmed by quantitative evaluation of the microscopic pictures and determination of the phagocytic index (Fig. 1D).

### The influence of AA was dose and time dependent.

**FIG 2:**
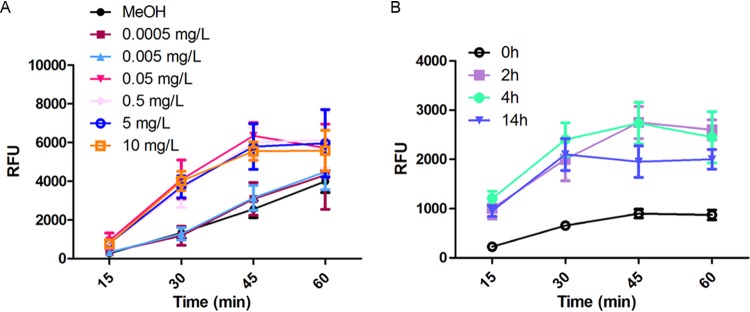
Influence of AA concentration and treatment time on the phagocytosis of C. albicans. (A) Influence of the AA concentration used for treatment of C. albicans on phagocytosis efficiency by the murine macrophage cell line RAW 264.7. (B) Influence of the AA (0.5 mg/liter) incubation time used for treatment of C. albicans on phagocytosis efficacy. Mean values and standard deviations were calculated from at least six biological replicates in each experiment, and the experiment was repeated 3 times. RFU, relative fluorescence units.

The first experiments with AA were performed with a single concentration, which had previously been proven to inhibit oxygen consumption and to induce ROS production. We now evaluated the influence of the AA concentration in a wider concentration range (Fig. 2A). We found that AA increased phagocytosis efficiency, when concentrations were higher than 50 μg/liter. There was no effect in the concentration range up to 5 μg/liter. This concentration range correlated with the range in which oxygen consumption was inhibited (data not shown). Moreover, we determined the phagocytosis efficiency after different points in time of AA treatment and found enhanced phagocytosis after 2 h of AA treatment (Fig. 2B), whereas shorter treatment periods led to no observable effects on phagocytosis efficacy but to almost instantaneous inhibition of oxygen consumption (data not shown). Thus, the effect on cell wall structure is not a primary effect of AA treatment but a secondary effect, which, however, is strongly linked to the inhibitory activity of AA on respiration.

We observed a slight inhibition of growth to approximately 90% at AA concentrations of 50 μg/liter and to 60% even at the highest AA concentrations, but the viability of the yeast, as indicated by propidium iodide (PI) staining, was not affected (data and methods are given in Fig. S1 in the supplemental material).

10.1128/mSphere.00016-20.1 FIG S1 Influence of the AA concentration on viability and growth of C. albicans. Mean values and standard deviations were calculated from three biological replicates in each experiment, and each experiment was repeated 3 times. **, *P* < 0.01. Download FIG S1, TIF file, 0.4 MB. Copyright © 2020 Cui et al. 2020 Cui et al. This content is distributed under the terms of the Creative Commons Attribution 4.0 International license.

### Treatment of C. albicans with AA increased the accessibility of cell wall glucans and decreased the amount of cell wall mannans.

β-(1,3)-Glucans are the major ligands for the C-type lectin-like receptor dectin-1, which is considered to be the main nonopsonic receptor to induce phagocytosis of C. albicans (16–18). Usually, β-(1,3)-glucans are covered by a mannan layer, thus minimizing recognition of C. albicans by dectin-1. However, it was previously shown that this 3-dimensional structure of the cell wall can be disturbed, which led to modified phagocytosis efficiencies (19). Thus, we investigated whether AA changed the cell wall structure and influenced the accessibility of β-(1,3)-glucans and mannans on the surface of C. albicans. β-(1,3)-Glucans were stained with a specific antibody, mannans were stained with concanavalin A, and binding was evaluated by flow cytometry.

**FIG 3:**
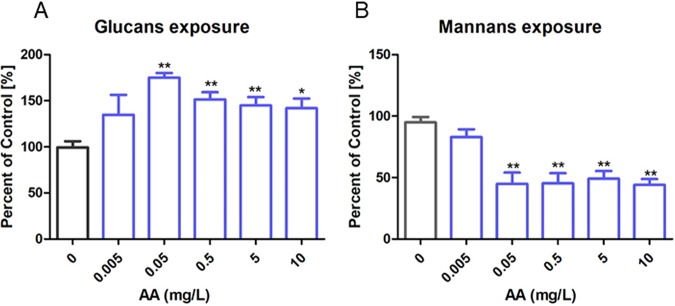
Influence of AA concentration on the accessibility of β-(1,3)-glucans and mannans of the cell wall of C. albicans analyzed by flow cytometry. The median fluorescence intensities of untreated cells were set as 100%. The percentages of median fluorescence intensities for different concentrations of AA treatment compared with untreated cells are given. Accessibilities of β-(1,3)-glucans (A) and mannans (B) from the cell wall of C. albicans treated with AA were analyzed by flow cytometry. For β-(1,3)-glucans, yeasts were probed with an anti-β-1,3-glucan antibody followed by incubation with an Alexa Fluor 488-labeled secondary antibody. For mannans, yeasts were probed with concanavalin A-FITC. The results from 4 independent experiments were analyzed. **, *P* < 0.01; *, *P* < 0.05.

In Fig. 3, the influence of AA concentrations on the median fluorescence intensity resulting from binding of fluorescently labeled anti-β-(1,3)-glucan antibodies and concanavalin A, respectively, is summarized. These fluorescence values are considered to correlate with the accessibilities of β-(1,3)-glucans and of mannans. In the concentration range up to 5 μg/liter, AA had no influence on β-(1,3)-glucan and mannan accessibilities, whereas when AA concentrations were 50 μg/liter or higher, β-(1,3)-glucan accessibility increased, accompanied by a decrease of mannan exposure. These data are strongly correlated with the dose dependence of the enhancement of phagocytosis efficiency and proved the relevance of β-(1,3)-glucan exposure for phagocytosis efficiency. We confirmed by flow cytometric (FACS) analysis that the dectin-1 and Toll-like receptor 2 (TLR2) receptors were expressed on the cell surface of the macrophages (data not shown). Hence, we assume that after AA treatment accessible β-(1,3)-glucans were recognized by dectin-1 and phagocytosis was initiated.

### AA influenced the expression of mannosyltransferases and the cell wall thickness.

**FIG 4:**
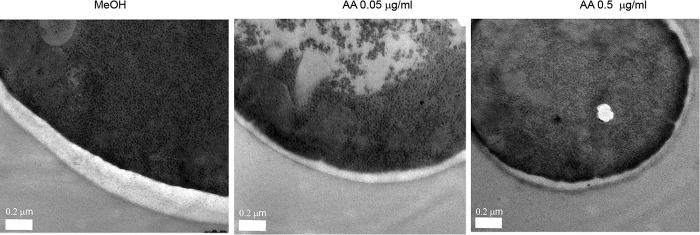
TEM images of the cell wall and cell membrane of C. albicans treated with AA for 24 h. Images were taken on a TEM at ×8,900 magnification. Cells without treatment showed a distinctive cell wall and cytoplasmic membrane. After treatment, the thickness of the cell wall and the integrity of the cell membrane are changed.

As AA treatment of C. albicans decreased the mannan accessibility (Fig. 3), we analyzed the influence of AA on the expression of genes which are involved in mannosylation of cell wall proteins (Table S1). AA treatment of only 20 min led to an upregulation of the expression of *MNN1* and *MNN22* and a downregulation of *VAN1*, *PMT1*, *MNT1*, and *GDA1* (Fig. S2). Thus, the observed downregulation of these genes after AA treatment is correlated with the reduced accessibility of mannans. Further, we analyzed the cell wall thickness in a rough view by transmission electron microscopy (TEM). The cell wall is significantly thinner after AA treatment in comparison to the control, as was also observed for the combination of sodium nitroprusside and SHA (10). Interestingly, the cell membrane was disrupted after AA treatment (Fig. 4).

10.1128/mSphere.00016-20.2 FIG S2 Influence of AA on the expression of C. albicans genes involved in protein mannosylation analyzed by RT-PCR. Cells were treated with AA (1.5 mg/liter) for 20 min. Mean values and standard deviations were calculated from three independent experiments. *, *P* < 0.05. Download FIG S2, TIF file, 1.1 MB. Copyright © 2020 Cui et al. 2020 Cui et al. This content is distributed under the terms of the Creative Commons Attribution 4.0 International license.

10.1128/mSphere.00016-20.4 TABLE S1 Genes and primers used for RT-PCR. Download Table S1, DOCX file, 0.01 MB. Copyright © 2020 Cui et al. 2020 Cui et al. This content is distributed under the terms of the Creative Commons Attribution 4.0 International license.

### Oxidative stress did not influence cell wall structure.

**FIG 5:**
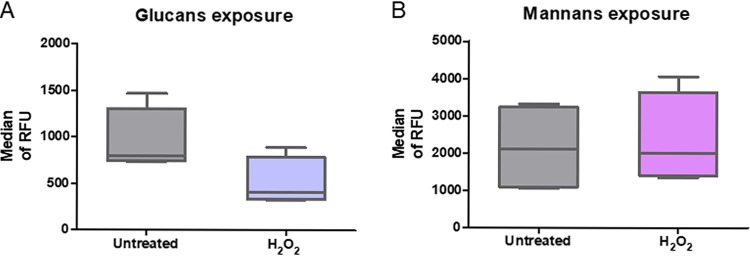
Influence of oxidative stress on the exposure of the cell wall component β-(1,3)-glucans and mannans. Accessibilities of β-(1,3)-glucans (A) and mannans (B) from the cell wall of C. albicans treated with H_2_O_2_ (2 mM) were analyzed by flow cytometry. For β-(1,3)-glucans, yeasts were probed with an anti-β-1,3-glucan antibody followed by incubation with an Alexa Fluor 488-labeled secondary antibody. For mannans, yeasts were probed with concanavalin A-FITC. The box plots show the median of RFU for 4 independent experiments. The bottom and top of the box represent the lower and upper quartiles, respectively. The band inside the box shows the median of RFU for 4 independent experiment. Whiskers display minimum and maximum of all data.

Inhibition of complexes of the respiratory chain, particularly of complex I and complex III, strongly induces the production of reactive oxygen species (ROS) (19), and a role for ROS in β-glucan masking was shown by studies with *sod1Δ* and *aox1Δ* mutants under hypoxic conditions (11). Thus, we wondered whether oxidative stress was also involved in the AA effects on the cell wall structure. We used H_2_O_2_ to expose C. albicans to oxidative stress; the H_2_O_2_ concentration (2 mM) was chosen on the basis of literature information (20). This concentration had no influence on the C. albicans viability, which was experimentally confirmed (data not shown). As can be seen in Fig. 5A and B, the cell wall structure did not significantly change when C. albicans was treated with H_2_O_2_, as no decrease in mannan accessibility was detected, and β-(1,3)-glucan accessibility was even slightly decreased. Moreover, no influence on the phagocytosis efficiency was observed (data not shown). Thus, the influence of AA on C. albicans could not be mimicked by oxidative stress generated by hydrogen peroxide.

### AA treatment changed the metabolite profile.

Determination of selected metabolites in yeast cultures with respiratory chain inhibitors (21) had shown that energy metabolism is related to the primary metabolism, inducing, for example, fermentative pathways. Proteomic and transcriptomic profiling of C. albicans mutants affecting the functionality of complex I (22) highlighted that the effects on metabolism go even beyond these pathways but might affect also the cell wall and the plasma membrane compositions. That is why we evaluated the effect of AA on the intracellular metabolic profiles of C. albicans by gas chromatography-mass spectrometry (GC-MS) analysis. We chose an AA concentration of 1.5 mg/liter and an incubation time of 4 h for these experiments, as these were conditions which had reproducibly led to the enhancement of phagocytosis and β-(1,3)-glucan accessibility and decrease of mannan exposure.

**FIG 6:**
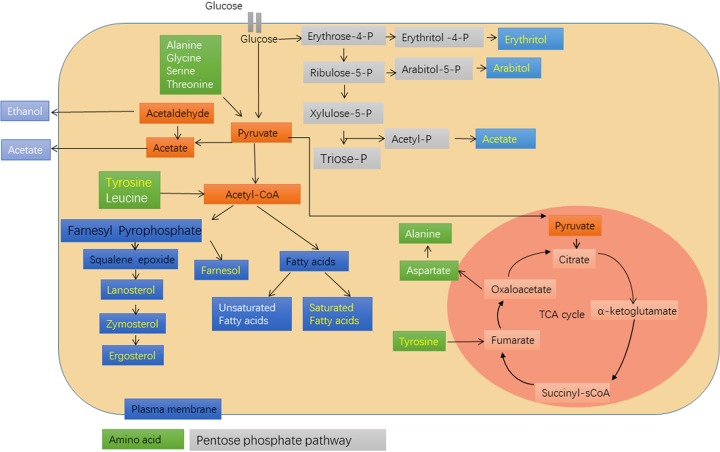
Scheme of AA on the metabolism pathways of C. albicans. Glucose penetrates the plasma membrane and is metabolized by glycolysis and converted into pyruvate and acetyl-CoA in the cytosol. Acetyl-CoA is involved in the biosynthesis of ergosterol and farnesol and fatty acids. Moreover, glucose is fed in the pentose phosphate pathway, and erythritol, arabitol, and acetate are synthesized. Also, pyruvate enters the mitochondrion and initiates the TCA cycle; alanine and aspartate are produced. Letters in yellow show downregulation, whereas letters in white show upregulation.

In the principal-component analysis (PCA) score plot, each point represented a sample and each cluster represented a corresponding metabolic pattern in different groups. A clear separation of the metabolic profiles of the control group (black dots) and of the AA group (red dots) was observed (Fig. S3A). The S plot (Fig. S3B), considering both the covariance P1 and correlation Pcorr loading proﬁles resulting from the orthogonal partial least squares discrimination analysis (OPLS-DA) model, visualizes the variable inﬂuence in a model, thus ﬁltering interesting metabolites in the projection. The metabolites which were significantly decreased in the AA group were in the lower left quadrant (negative correlation and covariance), and the increased ones were in the upper right quadrant (positive correlation and covariance). A loading plot, colored according to the absolute correlation coefﬁcients (|*r*|) of each variable to class separation (red means a high value; blue shows a low value), provided additional information on the metabolic variations among groups. Negative regions in the loading plot (Fig. S3B and C) corresponded to metabolites that were increased in the AA group; conversely, positive regions corresponded to metabolites that were decreased in the AA group. Forty metabolites were identified, of which 18 metabolites showed signiﬁcant concentration changes in response to AA (Table 1, Fig. 6, and Table S2). Functional analysis revealed that, particularly, amino acid biosynthesis, the ergosterol biosynthesis pathway, and fatty acid metabolism (e.g., biosynthesis of unsaturated/saturated fatty acids) were affected by treatment with AA. All major metabolites from the ergosterol biosynthesis pathway (ergosterol, ergostatetraenol, zymosterol, and lanosterol) and also farnesol could be identified, and most of them were significantly downregulated by AA treatment, as also observed by Grahl et al. (23). Moreover, concentrations of saturated fatty acids (C_12_, C_14_, and C_20_) were decreased, which was in line with lower concentrations of metabolites related to the pentose phosphate pathway, arabitol and erythritol. On the other hand, the concentrations of unsaturated fatty acids (palmitelaidic acid, octadecadienoic acid, and linolenic acid) increased, probably as a consequence of the oxidative stress. These data clearly show that the composition of the cell membrane changed as a result of AA treatment.

**TABLE 1:**
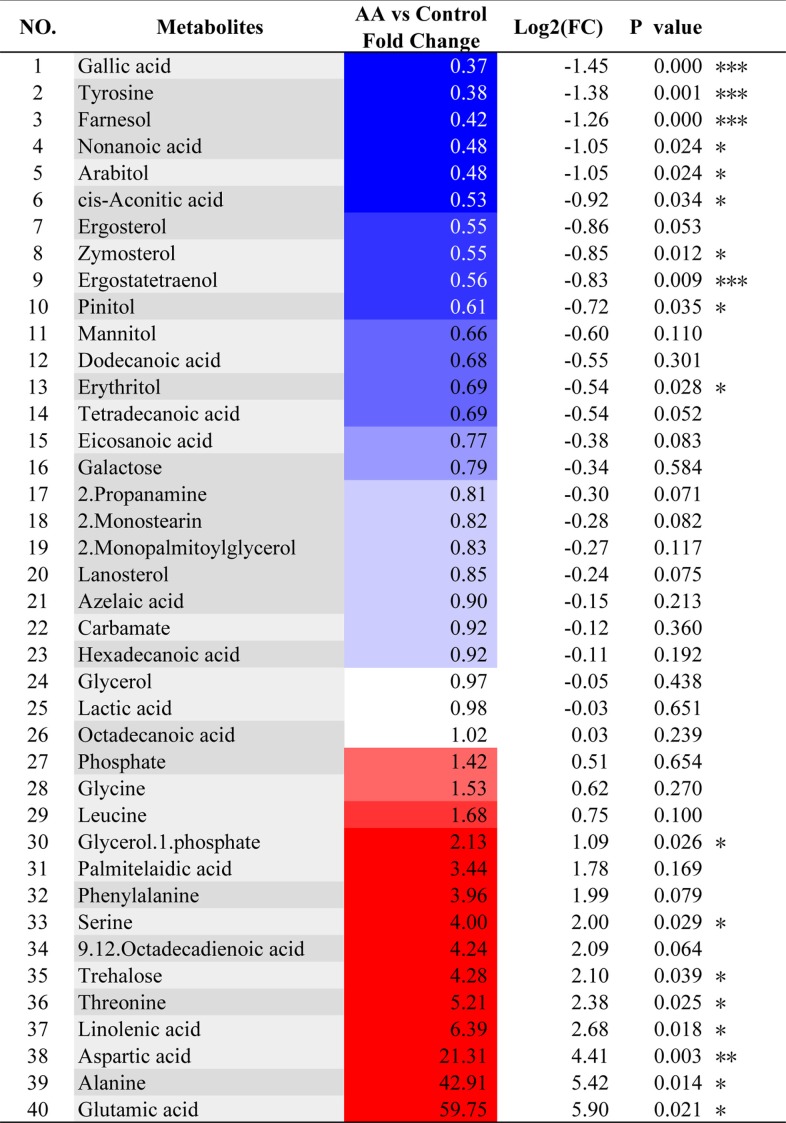
Important metabolite assignments in C. albicans, their fold change values, and associated *P* values^*a*^

a Color coded according to the log_2_(fold): red and blue represent increased and decreased metabolites, respectively, in the AA-treated group. *P* values were calculated based on a parametric Student *t* test. Numbers of asterisks denote extent of significance: *, *P* < 0.05; **, *P* < 0.01; ***, *P* < 0.001.

10.1128/mSphere.00016-20.3 FIG S3 OPLS-DA of GC-MS data from C. albicans. (A) PCA score plot. Component 1 and component 2 explained 86.6% of total variances in the extracts of C. albicans samples. (B) S-plot. (C) Color-coded coefficient loading plots. The color bar was applied in which red and blue represented metabolites that statistically signiﬁcantly or indistinctively contributed to the separation of groups, respectively. Peaks in positive and negative status revealed decreased and increased metabolites relative to score plot in the antimycin-treated group, respectively. Download FIG S3, TIF file, 0.6 MB. Copyright © 2020 Cui et al. 2020 Cui et al. This content is distributed under the terms of the Creative Commons Attribution 4.0 International license.

**FIG 7:**
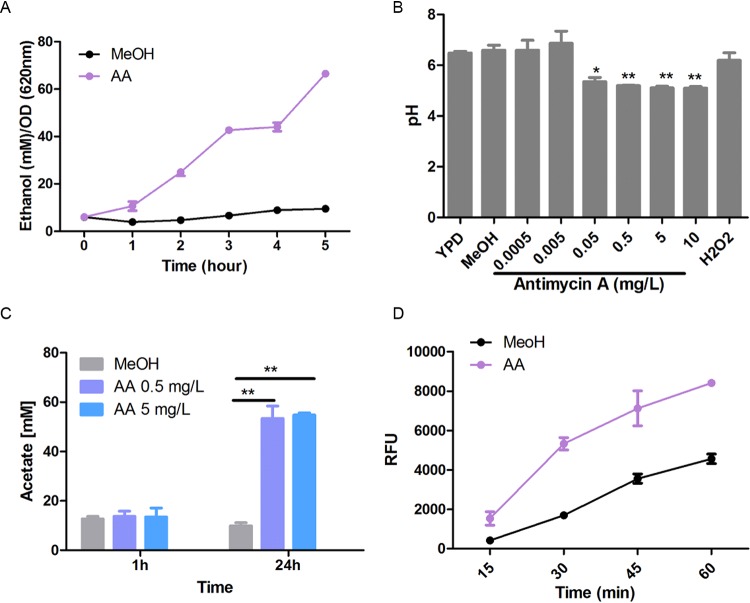
Effect of extracellular metabolites and pH change of AA-treated C. albicans. (A) Effect of AA (1.5 mg/liter) on ethanol production by C. albicans. The supernatant of AA-treated C. albicans was collected every 1 h. Ethanol concentrations were determined by enzymatic assays. (B) Effect of AA (1.5 mg/liter ) and H_2_O_2_ (2 mM) on the pH change of the C. albicans culture. The supernatant from overnight culture was collected, and the pH value of the cultivation medium was measured with a pH meter. (C) Effect of AA on acetate production by C. albicans. The supernatant of C. albicans treated with AA for 1 h and 24 h was collected, and the acetate concentration was detected by HPLC. (D) Phagocytic effect of AA (0.5 mg/liter) on C. albicans grown in MOPS-buffered YPD medium (pH 7.3). Mean values and standard deviations were calculated from three replicates in each experiment, and each experiment was repeated 3 times. **, *P* < 0.01; *, *P* < 0.05.

The increased concentrations of most of the identified amino acids, in particular of aspartic acid, glutamic acid, and alanine, probably reflect the high activity of glycolysis and the citric acid cycle, to compensate for the lack of energy generation through the electron transfer chain (ETC). The generated reducing equivalents (NADH) have to be reoxidized to allow glycolysis to proceed, leading to the secretion of fermentation products. As we had previously observed ethanol production as a result of complex III inhibition (21), we measured ethanol and acetic acid, an accompanying metabolite of ethanol production, in the supernatant of the AA-treated C. albicans cultures. Both ethanol (Fig. 7A) and acetate (Fig. 7C) concentrations were significantly increased. The latter correlated with a decrease of the pH to approximately pH 5 (Fig. 7B). The influence of the AA concentration on the pH of the supernatants correlated with the dose effect on the phagocytosis efficiency, so that we wondered whether the decreased pH might be the reason for the observed AA effects. Thus, AA treatment was repeated in a morpholinepropanesulfonic acid (MOPS)-buffered medium (pH 7.3), so that the pH did not change during overnight cultivation. When these cells were used in the phagocytosis assay, we still observed the increased phagocytosis efficiency (Fig. 7D).

## DISCUSSION

Mitochondria play an important role for cell growth, metabolism, and virulence of Candida albicans. However, they prove to be a central hub for C. albicans physiology as their functionality affects not only the energy metabolism and central carbon metabolism but also the biosynthesis of constituents of the cell membrane and the cell wall and the susceptibility to drugs. Thus, inhibition of mitochondrial functions is nowadays discussed as a new target for antifungals (14, 24–27). Previous studies were mainly focused on fungus-specific proteins, particularly those affecting the activity of complex I of the respiratory chain.

Compared to these studies, we were mainly interested in manipulating the cell wall structure by specific inhibitors, because the increased phagocytosis efficacy of macrophages for some C. albicans deletion mutants was already shown and we wanted to obtain similar effects by chemical treatment of C. albicans. Thus, as a proof of concept we cultivated C. albicans in the presence of inhibitors of single complexes of the electron transport chain and first studied the interaction with macrophages. We could now unambiguously show that particularly treatment with the complex III inhibitor antimycin A improved phagocytosis efficacy. In subsequent studies, we found a good correlation between concentrations required for respiration inhibition and those for the stimulatory effect on phagocytosis. However, whereas fungal oxygen consumption was almost immediately blocked by the presence of AA, the effects on phagocytosis required extended incubation times of at least 2 h, showing that these effects were secondary effects of AA treatment. To elucidate the underlying mechanisms, we analyzed the cell wall structure and also the cellular metabolism. AA treatment changed the cell wall structure as it leads to increased exposure of β-(1,3)-glucans and decreased accessibilities of mannans. Similar results were recently reported in which respiration of C. albicans was inhibited by the combined addition of the nitric oxide donor sodium nitroprusside and the inhibitor of alternative oxidase SHA (10). Deeper analysis of the effects on the intracellular metabolome showed increased synthesis of some amino acids, a phenomenon also reported for the *goa1Δ* mutant (22), and a significant downregulation of metabolites from the mevalonate pathway. This pathway leads from acetyl coenzyme A (acetyl-CoA) via mevalonate to farnesyl pyrophosphate (FPP), where it branches to farnesol and farnesylated proteins, such as Ras1 (28), and to ergosterol biosynthesis (Fig. 6). The downregulation may be due to the consumption of pyruvate in the activated tricarboxylic acid (TCA) cycle and in fermentation reactions to ensure sufficient energy production and the redox balance. This hypothesis is supported by the observation that ergosterol pathway downregulation is not specific for complex III inhibition but was also described for the *ndh51Δ* mutant (26). The need for alternative energy sources can also be the reason for the low levels of saturated fatty acids, feeding additional acetyl-CoA into the TCA cycle via β-oxidation. The increased levels of unsaturated fatty acids, however, could be due to the presence of high ROS levels and the resulting oxidation of fatty acids by desaturases. Overall, treatment of C. albicans with AA not only resulted in the known increased concentrations of ROS and fermentation products but led to significant redirections of metabolic pathways which influenced the composition of the cellular membrane and thus its fluidity and permeability. This was supported by the TEM results showing defects in the cell membrane of AA-treated yeast. As cell wall components, such as proteins and carbohydrate chains, have to be transported through the cell membrane, changes in the membrane composition may be the reason for the observed changes of the cell wall structure, which enhanced the phagocytosis efficiency of macrophages. The relevance of lipids and lipid biosynthesis for the cell wall was also reported by Dagley et al. (27) and was related to the functionality of membrane complexes, such as the SAM37 complex and the ERMES complex, which are essential for protein transport and interactions between organelles.

Thus, with the current study we could highlight a specific role of complex III of the respiratory chain for the cell wall structure, which was not previously shown. The results could be exploited by the search for fungus-specific inhibitors of this complex to inhibit one of the immune escape mechanisms of C. albicans.

## MATERIALS AND METHODS

### Media and reagents.

Dulbecco’s modified Eagle’s medium (DMEM) and fetal bovine serum (FBS) were from Lonza (Verviers, Belgium). YPD medium (yeast extract [10 g/liter], peptone [20 g/liter], and glucose [20 g/liter]), MOPS-buffered YPD medium (YPD medium plus MOPS 165 mM), yeast nitrogen base (YNB) medium, 100× penicillin, and streptomycin were purchased from Sigma (Saint Louis, MO, USA). Dimethyl sulfoxide (DMSO) was from Biomol GmbH (Hamburg, Germany). The inhibitors antimycin A (AA), rotenone, and salicylhydroxamic acid (SHA) were from Sigma, thenoyltrifluoroacetone (TTFA) was from Fluka (Saint Louis, MO, USA), and potassium cyanide (KCN) and H_2_O_2_ were from Roth (Karlsruhe, Germany) and Merck (Darmstadt, Germany), respectively. Anti-β-1,3-glucan antibodies were from Biosupplies (Parkville, Australia). MOPS, fluorescein isothiocyanate (FITC), FITC-labeled concanavalin A (FITC-ConA), propidium iodide (PI), and the formalin solution were purchased from Sigma, and stock solutions were prepared: 1 mg/ml in 0.9% NaCl for FITC-ConA, 100 mg/ml in dimethyl sulfoxide (DMSO) for FITC, and 20 mM for propidium iodide also in DMSO. Trypan blue was from Fluka.

### Cell culture.

The murine macrophage cell line RAW 264.7 was purchased from the American Type Culture Collection (Manassas, VA). The cells were grown in DMEM supplemented with 10% fetal bovine serum (FBS), 100 U/ml penicillin, and 100 μg/ml streptomycin at 37°C in a 5% CO_2_-in-air atmosphere.

### Microorganisms and culture conditions.

If not mentioned otherwise, growth was followed via determination of optical density at 620 nm (OD_620_) in a 180-μl sample volume using the μQuant microplate reader (BioTek Instruments, Bad Friedrichshall, Germany).

C. albicans strains SC5314 (ATCC MYA-2876) and CAF2-1 (29) were cultivated overnight in 100-ml flasks in 20 ml of YPD medium at 30°C with orbital shaking at 160 rpm. From this overnight culture, a preculture was prepared by dilution to an OD_620_ of 0.2 in 25 ml YPD medium.

The cells were allowed to grow for 3 h so that they reached the exponential growth phase. The working culture was prepared by diluting the preculture to an OD_620_ of 0.1 in 25 ml YPD medium supplemented with or without electron transfer chain inhibitor. The cultures were allowed to grow at 30°C with orbital shaking at 160 rpm overnight. The final concentrations of electron transfer chain (ETC) inhibitors were 40 mg/liter of rotenone, 5 mg/liter of TTFA, 50 mg/liter of SHA, 0.5 mg/liter of AA, and 5 mg/liter of KCN. The concentrations were chosen according to previous studies (15) and were based on inhibitory effects on oxygen consumption and reactive oxygen species (ROS) induction.

For fluorescence labeling, 1 × 10^8^ yeast cells were harvested by centrifugation (13,000 rpm, 5 min, 24°C), washed twice with 1 ml phosphate-buffered saline (PBS), and stained with 1 ml of 500 mg/liter FITC at 4°C overnight. Finally, yeast cells were carefully washed three times in PBS to remove excessive dye before use.

### Growth and viability of C. albicans.

C. albicans was cultivated overnight in YPD medium with and without AA and methanol as solvent control as described above. Growth of the cultures was estimated from measurements of optical densities at 620 nm. The investigations were independently performed three times with three samples per test.

The viability of the yeast cells was determined by PI staining. One milliliter of a yeast suspension was centrifuged at 10,000 × *g* for 3 min to pellet the cells. The supernatants were removed, and the cells were washed with PBS once and resuspended in 1 ml PBS. The samples were diluted to 10^6^ cells/ml, and 10 μl of the PI stock solution (2 mM) was added. Each sample was gently vortexed. The samples were incubated at room temperature or 37°C in the dark for 15 to 30 min. Cells were analyzed by flow cytometry without additional washing. Flow cytometric analysis was performed with a FACSCanto cytometer (Becton Dickinson, USA) (see Fig. S1 in the supplemental material).

### Phagocytosis assay.

Phagocytosis of C. albicans by macrophages was quantified as described previously (4). Briefly, 100 μl of 2 × 10^6^ macrophages/ml was seeded in each well of 96-well microplates (Nunc, Germany) followed by incubation for 2 h to let the cells adhere to the plates. The supernatant was replaced by 100 μl of a suspension of 4 × 10^6^ FITC-labeled yeasts in DMEM supplemented with 10% FBS, so that the ratio of macrophages to yeast was 1:2. Phagocytosis was allowed to proceed at 37°C in 5% CO_2_. At indicated time points, the yeast suspension was removed and 100 μl trypan blue (250 mg/liter in PBS) was added to quench the fluorescence of yeasts which were not internalized. After incubation at room temperature for 1 min, the trypan blue solution was removed. The number of internalized yeasts was estimated from fluorescence measurements (λ_Ex_ = 480 nm and λ_Em_ = 520 nm) through the bottom of the plates by a fluorescence microplate reader (Synergy 4; BioTek, Bad Friedrichshall, Germany).

Data analysis was based on the mean of the fluorescence values of eight wells per experiment, and each experiment was independently repeated at least twice. Background fluorescence was determined from the fluorescence of wells containing only FITC-labeled yeasts but no macrophages. Data are presented as the difference between the total fluorescence and the background.

As a control for the microplate assay, we investigated the ingestion of yeasts by fluorescence microscopy. Seven hundred fifty microliters of 5 × 10^5^ macrophages/ml was seeded in each of the 4 compartments of a Cellview glass-bottom dish (Greiner Bio-one, Frickenhausen, Germany) followed by incubation for 2 h to let the cells adhere to the plates. A C. albicans suspension was added at a 1:2 ratio of macrophages to yeast. Phagocytosis was allowed to proceed at 37°C in 5% CO_2_ for 1 h. Wells were washed with PBS and fixed with a 2% formalin solution. The numbers of macrophages and yeasts were recorded in arbitrarily chosen pictures, and at least 200 macrophages were counted. The phagocytosis index was defined as the ratio of the number of macrophages with intracellular yeasts to the total number of macrophages per picture.

### Detection of β-(1,3)-glucans and mannans on yeast cells.

β-(1,3)-Glucans were stained with specific Alexa Fluor 488-conjugated monoclonal antibodies (30). One milliliter of 10^6^ cells/ml was washed with PBS containing 2% FBS and then incubated with 200 μl of the anti-β-(1,3)-glucan antibody (1:150 dilution) for 1 h. Cells were washed in PBS containing 2% FBS 3 times and incubated with the secondary antibody (Alexa Fluor 488-labeled goat anti-mouse; 1:250 dilution) for 45 min. As a negative control, yeast cells were incubated only with the secondary antibody. After washing, cells were fixed in 0.4% formalin and analyzed by flow cytometry.

The detection of mannans was based on binding of concanavalin A (31). Suspensions from AA- and H_2_O_2_-treated C. albicans cultures were adjusted to an OD_620_ of 0.1 using sample volumes of 180 μl and the μQuant microplate reader. Cells were washed twice with 0.9% NaCl, collected by centrifugation, and resuspended in 500 μl of an FITC-concanavalin A solution (1:50 dilution with 0.9% NaCl of the stock solution of 1 mg/ml). After 30 min, cells were collected by centrifugation and washed twice with 1 ml 0.9% NaCl. After washing, cells were fixed in 0.4% formalin and analyzed by flow cytometry. All incubation and washing steps were done at room temperature.

### Gene expression analysis by real-time PCR (RT-PCR).

C. albicans was cultivated overnight in 250-ml flasks with 50 ml YPD medium at 30°C. A preculture was prepared by diluting the overnight culture in 25 ml YPD (OD_620_ of 0.2) and was cultivated for 3 h to reach the exponential growth phase. For the working culture, the preculture was diluted to an OD_620_ of 0.1 in YPD supplemented with or without 1.5 mg/liter AA. After cultivation at 30°C for 20 min, the cells were harvested by centrifugation, and the cell pellets were shock-frozen in liquid nitrogen. Frozen pellets were suspended in 0.6 ml RLT buffer (Qiagen) and mechanically disrupted using glass beads (425 to 600 μm; Sigma). RNA was isolated on RNeasy minicolumns with added DNase (Qiagen) as recommended by the manufacturer. The quality and integrity of total RNA of a subset of the samples were controlled with the Agilent Technologies 2100 Bioanalyzer (Agilent Technologies).

Three micrograms of total RNA was employed in reverse transcription, with Superscript II reverse transcriptase (RT) and random and oligo(dT)_12–18_ primers, according to the manufacturer’s recommendations (Invitrogen). Quantitative real-time PCR was carried out on a 96-well LightCycler 480 system using the LightCycler 480 SYBR green I master (Roche), as recommended by the manufacturer (95°C, 60°C, and 72°C for 10 s each for 45 cycles). Gene sequences were obtained from the C. albicans Genome Database (32), and gene-specific oligonucleotides (Table S1) were designed by Roche’s Probe Library Assay Design Center and synthesized by Eurofins MWG Operon. Specificity was controlled against the C. albicans genome sequence by using BLAST. Real-time analysis data (crossing points) were normalized with respect to the actin gene *ACT1*, and relative gene expression levels were calculated. The average and standard deviations of the gene expression levels relative to solvent controls in three independent experiments were calculated, and the significance of the changes in gene expression was tested by Student’s *t* test of normalized data (*P* < 0.05) (Fig. S2).

### Ultrastructural analysis of the cell walls of C. albicans after AA treatment using TEM.

Cells were treated according to the literature (33). Briefly, a suspension of C. albicans cells (1.5 × 10^8^ cells/ml) was prepared from yeast cultures grown for 24 h at 37°C in YPD with or without different concentrations of AA. The samples were centrifuged. The resultant pellets were each resuspended in 5 ml PBS. After washing two times, the fungal cells were fixed by resuspending each pellet in 1 ml of 4% formaldehyde and 1% glutaraldehyde in PBS. After 2 h of incubation at room temperature, the samples were stored at 4°C until they were stained with 1% osmium tetroxide (OsO_4_) at room temperature. After washing with PBS to remove excess OsO_4_, a dehydration series was performed with 25, 50, 75, 95, and 100% ethanol diluted in distilled water (dH_2_O). The samples were additionally dehydrated with propylene oxide, embedded in a resin, and left to harden for 48 h at 60°C. The resin capsules were cut using an ultramicrotome (Leica Ultracut UCT) and a 45° diamond knife. Ultrathin sections of approximately 95 nm were obtained and visualized using a CM100 TEM (Philips, Netherlands).

### Ethanol and acetate determination.

A C. albicans preculture was prepared as described above. Twenty-milliliter working cultures were prepared by diluting the preculture to an OD_620_ of 0.1 with YPD medium supplemented with 1.5 mg/liter AA or the respective amount of solvent (methanol). The sample volume was 200 μl of the cell suspension. Samples were taken every hour and immediately centrifuged at 14,000 rpm and 4°C for 10 min. Ethanol concentrations were determined by enzymatic assays based on ethanol dehydrogenase (21). Quantification was based on the photometric determination of NADH at 340 nm, and the assays were performed in volume-reduced 96-well transparent microplates (Corning). For acetate determination, C. albicans was grown with 0.5 and 5 mg/liter AA or the respective amount of solvent (methanol). Samples were taken after 1 h and 24 h. Acetate concentration was detected by high-performance liquid chromatography (HPLC) (Agilent, USA).

### pH measurement.

C. albicans was grown in YPD medium supplemented with or without various concentrations of AA at 30°C overnight. The suspension was collected by centrifugation at 5,000 rpm for 5 min, and the pH of the cultivation medium was determined with a pH meter.

### Extraction of intracellular metabolites.

C. albicans preculture was prepared as described above. Twenty-milliliter working cultures were prepared by diluting the preculture to an OD_620_ of 0.1 with YNB medium supplemented with 1.5 mg/liter AA or the respective amount of solvent (methanol) for 4 h. Six biological replicates were used for the treated group and the control group. The extraction of intracellular metabolites was done as described in literature (34, 35). Briefly: the cell pellet was obtained by 5 min of centrifugation (10,000 × *g*, 4°C), fixed with 60% cold MeOH in NaCl buffer, and washed with 5 ml cold phosphate-buffered saline (PBS) 3 times. The cell pellets were stored at −80°C and then lyophilized in a freeze drier.

The metabolic extract was prepared according to literature (36). Briefly, 100 μl of cell pellets was spiked with internal standard (15 μl heptadecanoic acid in water, 1 mg/ml) and vortexed for 2 min. The mixed solution was extracted with 400 μl methanol and centrifuged for 10 min at a rotation speed of 13,000 rpm at 4°C. An aliquot of 450 μl of the supernatant was transferred to a clean glass sampling vial and dried under a gentle stream of nitrogen at room temperature. For derivatization, 80 μl methoxamine hydrochloride (20 mg/ml in pyridine) was added to the vial and kept at 60°C for 4 h followed by the addition of 60 μl of 1% *N*-methyl-*N*-(trimethylsilyl)trifluoroacetamide (MSTFA; 1% TMCS) for 1 h at 70°C. An aliquot of 2 μl of the derivatized sample was injected into an Agilent 7890B gas chromatograph coupled with an Agilent 5977A mass spectrometer. Separation was performed on an HP-5MS capillary column (30 m by 0.25-mm inside diameter [i.d.], 0.25-μm film thickness; Agilent, USA) with He as the carrier gas at a constant flow rate of 1 ml/min. The solvent delay time was set to 5 min. The temperatures of injection, transfer interface, and ion source were 250°C, 290°C, and 230°C, respectively. The GC temperature programming was set to 2 min of isothermal heating at 80°C, followed by 5°C/min oven temperature ramps to 240°C and 8°C/min to 300°C, and a final 6-min maintenance at 300°C. Ionization mode was electron ionization (EI) mode with the electron energy of 70 eV. A full scan mode with a scannning range of *m/z* 50 to 600 was used.

### Data analysis.

The GC-MS raw data were converted to an mzXMLfile and then imported into R software (http://cran.r-project.org/) for noise filtering, baseline correction, and ion peak alignment. The data were then probability quotient normalized and analyzed by orthogonal signal correction partial least squares discriminant analysis (OSC-PLS-DA) to differentiate each group. The OSC-PLS-DA model was cross-validated using a 7-fold method by default; the validity of the models against overﬁtting was assessed by the parameters *R*^2^*Y*, and the predictive ability was described by *Q*^2^*Y* (37). Negative or very low *Q*^2^*Y* values indicate that the differences between groups are not statistically signiﬁcant. Metabolites were identified by comparing the mass fragmentations with NIST 05 standard mass spectral database (National Institute of Standards and Technology [NIST], Gaithersburg, MD) with a similarity of more than 70% and finally verified by available reference standards.

### Statistics.

Diagrams were created with Excel 2010 and GraphPad Prism 8 (GraphPad Software, San Diego, CA, USA), respectively. For comparative statistical analysis, Student’s unpaired *t* test was performed using Microsoft Excel 2010. *P* values of <0.05 were considered significant.

### Data availability.

All data have been provided in the paper and in the supplemental material.

10.1128/mSphere.00016-20.5 TABLE S2 Integration area of metabolites in the GC-MS spectra. Download Table S2, CSV file, 0.01 MB. Copyright © 2020 Cui et al. 2020 Cui et al. This content is distributed under the terms of the Creative Commons Attribution 4.0 International license.
