# Supplementary figures and images for: Inhibition of Respiration of Candida albicans by Small Molecules Increases Phagocytosis Efficacy by Macrophages
Source: mSphere. 2020 Apr 15;5(2):e00016-20. doi: 10.1128/mSphere.00016-20 (PMC7160677; doi:10.1128/mSphere.00016-20)

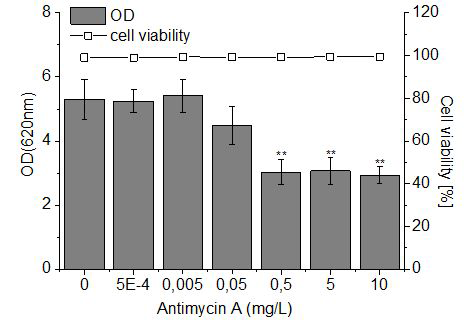

Supplement: FIG S1 [file mSphere.00016-20-sf001.tif]

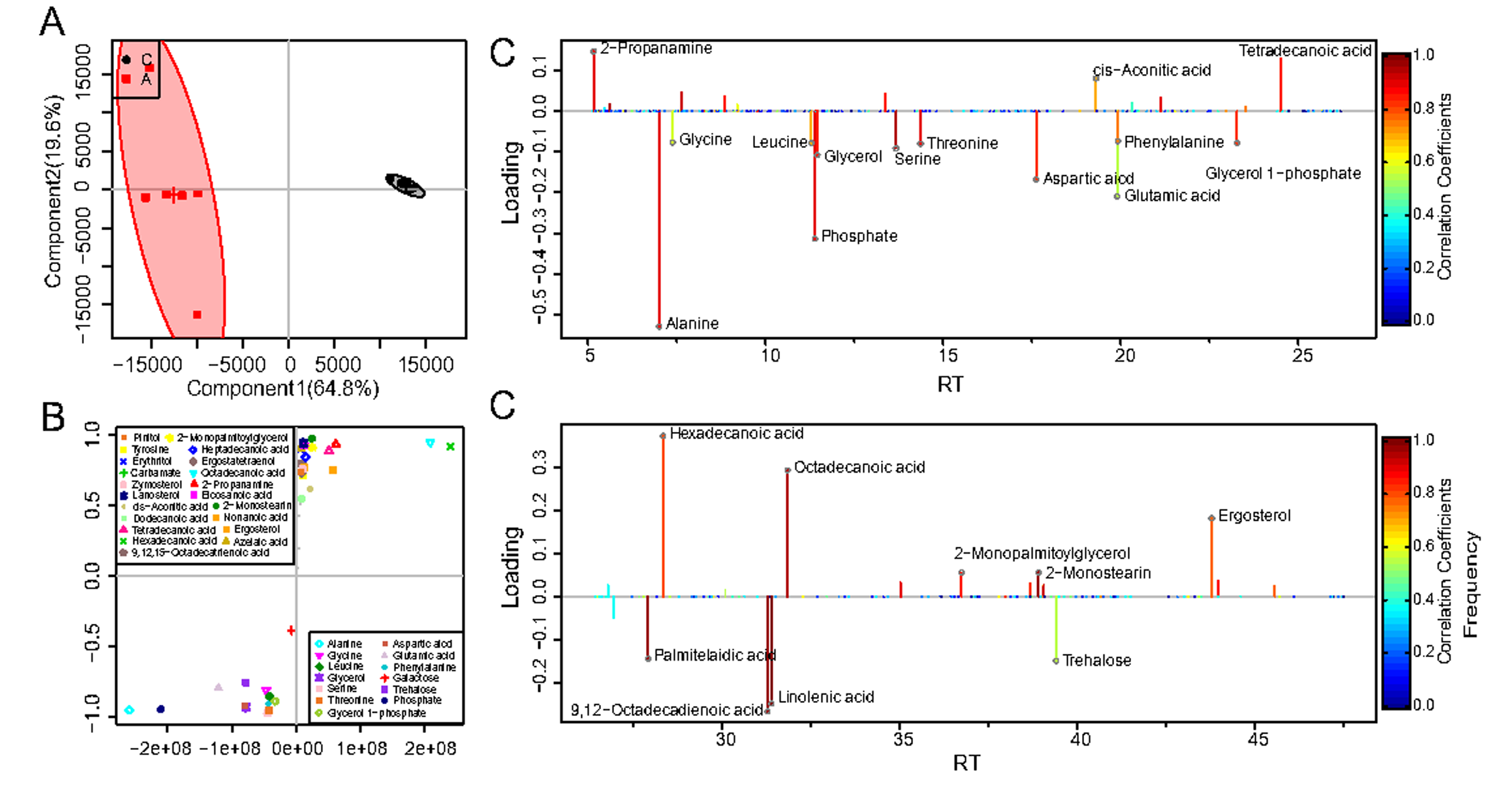

Supplement: FIG S3 [file mSphere.00016-20-sf003.tif]
