# Supplementary material for: Inhibition of Respiration of Candida albicans by Small Molecules Increases Phagocytosis Efficacy by Macrophages
Source: mSphere. 2020 Apr 15;5(2):e00016-20. doi: 10.1128/mSphere.00016-20 (PMC7160677; doi:10.1128/mSphere.00016-20)
Supplement: TABLE S1 [file mSphere.00016-20-st001.docx]

Table 1: Genes and primers used for RT-PCR

| Target gene | Protein function according toCandida Genome Database | **Oligonucleotide** | |
| --- | --- | --- | --- |
|  |  | Name | Sequence |
| *CaACT1* | Actin | *CaACT1*left | aaccaccggtattgttttgg |
|  |  | *CaACT1*right | gcgtaaattggaacaacgtg |
| *CaMNN1* | Putative α-1,3-mannosyltransferase | *CaMNN1*left | catttacacacagcagaccagtt |
|  |  | *CaMNN1*right | ccatagcatttttacgatcttgc |
| *CaMNN22* | Putative Golgi α-1,2-mannosyltransferase | *CaMNN22*left | aacagataatgaccccaatgaga |
|  |  | *CaMNN22*right | ttttggtcacaaagaatctcagaa |
| *CaVAN1* | Member of Mnn9p family of  Mannosyltransferases | *CaVAN1*left | tgtgtggagacctttacctacatt |
|  |  | *CaVAN1*right | agcatctgattcaacccaaga |
| *CaPMT1* | Protein mannosyltransferase (PMT), one of 5 PMT family members | *CaPMT1*left | caatgaaaacagtatattgcctcct |
|  |  | *CaPMT1*right | ttgccaaagggataatttaggat |
| *CaMNT1* | α-1,2-mannosyltransferase, adds second mannose during cell-wall mannoprotein biosynthesis | *CaMNT1*left | ccgctcatgaaaaacagacc |
|  |  | *CaMNT1*right | ctgcggaacctgaaccag |
| *CaGDA1* | Golgi membrane GDPase, required for wild-type O-mannosylation | *CaGDA1*left | tgctgctgcaccagtgtc |
|  |  | *CaGDA1*right | tggcttccttcttgttgctt |
